# Off-Line SPE LC-LRMS Polyphenolic Fingerprinting and Chemometrics to Classify and Authenticate Spanish Honey

**DOI:** 10.3390/molecules27227812

**Published:** 2022-11-13

**Authors:** Víctor García-Seval, Javier Saurina, Sònia Sentellas, Oscar Núñez

**Affiliations:** 1 Department of Chemical Engineering and Analytical Chemistry, University of Barcelona, Martí i Franquès 1-11, E08028 Barcelona, Spain; 2 Research Institute in Food Nutrition and Food Safety, University of Barcelona, Recinte Torribera, Av. Prat de la Riba 171, Edifici de Recerca (Gaudí), Santa Coloma de Gramenet, E08921 Barcelona, Spain; 3 Serra Húnter Fellow, Generalitat de Catalunya, Via Laietana 2, E08003 Barcelona, Spain

**Keywords:** blossom honeys, honeydew honeys, off-line SPE, polyphenols, LC-LRMS, fingerprinting, chemometrics

## Abstract

The feasibility of non-targeted off-line SPE LC-LRMS polyphenolic fingerprints to address the classification and authentication of Spanish honey samples based on both botanical origin (blossom and honeydew honeys) and geographical production region was evaluated. With this aim, 136 honey samples belonging to different botanical varieties (multifloral and monofloral) obtained from different Spanish geographical regions with specific climatic conditions were analyzed. Polyphenolic compounds were extracted by off-line solid-phase extraction (SPE) using HLB (3 mL, 60 mg) cartridges. The obtained extracts were then analyzed by C18 reversed-phase LC coupled to low-resolution mass spectrometry in a hybrid quadrupole-linear ion trap mass analyzer and using electrospray in negative ionization mode. Principal component analysis (PCA) and partial least squares-discriminant analysis (PLS-DA) were employed to assess the pattern recognition capabilities of the obtained fingerprints to address honey classification and authentication. In general, a good sample discrimination was accomplished by PLS-DA, being able to differentiate both blossom-honey and honeydew-honey samples according to botanical varieties. Multiclass predictions by cross-validation for the set of blossom-honey samples showed sensitivity, specificity, and classification ratios higher than 60%, 85%, and 87%, respectively. Better results were obtained for the set of honeydew-honey samples, exhibiting 100% sensitivity, specificity, and classification ratio values. The proposed fingerprints also demonstrated that they were good honey chemical descriptors to deal with climatic and geographical issues. Characteristic polyphenols of each botanical variety were tentatively identified by LC-MS/MS in multiple-reaction monitoring mode to propose possible honey markers for future experiments (i.e., naringin for orange/lemon blossom honeys, syringic acid in thyme honeys, or galangin in rosemary honeys).

## 1. Introduction

Honey is a very sweet and viscous fluid foodstuff produced by domestic bees of the genus Apis (*Apis mellifera*). There are two main types of honey, depending on the substance employed by the bees as source: blossom honeys (or nectar honey), when bees use the nectar of plant flowers, and honeydew honeys, when they use the secretions of plants or excretions produced by plant-sucking insects. These substances collected by the bees are then transformed into honey by combining them with other substances provided by the bees or generated through the honey-maturation process in the hives by means of biochemical reactions [1,2,3,4]. Honey is a very appreciated and highly consumed food product that has been used for thousands of years throughout the world, either as a natural sweetener or as a flavoring for concoctions, because of its taste and nutritional value, but also as traditional medicine due to its health benefits (i.e., antioxidant, anti-inflammatory, antifungal, and antibacterial effects) [5,6,7,8,9,10]. Its composition is quite variable but the main components are carbohydrates (about 76%), in the form of fructose and glucose, disaccharides, such as maltose, isomaltose, sucrose, maltulose, turanose, and nigerose, as well as oligosaccharides, such as panose. In addition, it also contains enzymes (amylase, peroxide oxidases, and catalase, among others), amino acids, vitamins (ascorbic acid, niacin, riboflavin, and pantothenic acid), minerals (calcium, magnesium, phosphorus, manganese, zinc, iron, and potassium), and bioactive substances, such as polyphenols, responsible for the antioxidant properties [11,12,13,14,15].

Polyphenols are aromatic secondary metabolites of plants that are generally involved in their defense mechanisms against aggressions by pathogens, hydric stress, and ultraviolet radiation [16]. In food products, polyphenols contribute to color, flavor, odor, astringency, and oxidative stability. They can be divided into several classes, depending on their structures: phenolic acids (hydroxybenzoic and hydroxycinnamic acids), flavonoids, stilbenes, and lignans. In addition to their contribution to organoleptic and nutritional properties in food products, including honey, their presence and distribution can also be employed as markers to characterize and differentiate among different honey botanical varieties, especially between blossom and honeydew honeys [1,17,18]. Phenolic acids and flavonoids are the two families of polyphenolic compounds more widely found in honey samples.

Depending on the plant source employed by the bees, honeys can be considered a polyfloral or multifloral (MF) variety, when multiple botanical sources are present, or monofloral variety when either the whole product or most of it comes from a specific botanical source, prevailing the microscopic, physicochemical, and organoleptic properties of that botanical source. However, there are still several discrepancies regarding the minimum content of a botanical source necessary to consider a type of honey as monofloral. The minimum pollen percentage required depends on each country’s legislation, 45% being normally accepted, although this level changes for some specific botanical varieties [3,4,19,20]. The assessment of both botanical source and geographical origin becomes an important issue for society because of the differences in honey properties and price. For example, honey produced within European countries tends to be considered of higher quality and, accordingly, more expensive than those obtained from China. In this context, honey is one of the food products most susceptible to fraudulent practices, for instance, by mislabeling or by adding sugar-based adulterants, such as syrups or sugar-based products, with the aim of obtaining an economic benefit [21,22,23]. Hence, feasible analytical methodologies to characterize, classify, and authenticate honey are needed to fight against this kind of fraud.

Both targeted and non-targeted analytical methodologies have been applied to address honey authenticity issues. In targeted strategies, selected compounds or families of compounds with similar structural or physicochemical properties are monitored as markers for classification and authentication purposes [24]. The analytical method is, therefore, optimized to enhance extraction performance and facilitate the discrimination of these compounds from the analyzed sample matrix. For example, volatile organic compounds have been used to differentiate between monofloral honeys from different floral sources, thus, providing valuable information concerning the honey botanical and geographical origins [25]. The analysis of sugars is also a successful targeted strategy to detect adulterations made with sugar syrups to increase bulk volume [26]. Bioactive substances, such as phenolic acids and flavonoids, are also widely employed for the characterization, classification, and authentication of honeys of different botanical and geographical origins [17,27,28,29]. The main drawback of targeted methodologies is that chemical standards are required for quantitation purposes or to guarantee compounds’ identity, which can be a time-consuming and difficult task when analyzing complex matrices. In contrast, non-targeted strategies rely on rich sample fingerprints, consisting of registering as many instrumental responses as possible, but without the requirement of knowing the compounds responsible for the obtained responses. The monitoring of direct spectral information by means of near-infrared, ultraviolet-visible, or fluorescence spectroscopy, for example, has been used to address honey authentication issues [30,31,32]. Liquid chromatographic techniques either with UV detection [33,34] or coupled to low-resolution mass spectrometry (LC-MS) or high-resolution mass spectrometry (LC-HRMS) have also been widely employed in non-targeted fingerprinting (metabolomic) strategies for honey authentication [35].

In a previous study, we developed a non-targeted HPLC-UV fingerprinting strategy for the classification of honey samples by employing an off-line solid-phase extraction (SPE) sample treatment and a C18 chromatographic separation, specifically designed for the isolation and separation of honey polyphenolic compounds [34]. Although discrimination between blossom and honeydew honeys was accomplished when non-targeted off-line SPE HPLC-UV fingerprints were used as sample chemical descriptors, no discrimination was accomplished within the different honeydew-honey botanical varieties under study nor when addressing the honey geographical production region. The present contribution aimed to improve the discrimination capability of the proposed off-line SPE polyphenolic fingerprints by employing liquid chromatography coupled to low-resolution mass spectrometry in a hybrid quadrupole-linear ion trap mass analyzer working in full scan and using electrospray in negative ion mode as the ionization source. The feasibility of the obtained non-targeted off-line SPE LC-LRMS fingerprints to address honey classification and authentication based on botanical and geographical production region by principal component analysis (PCA) and partial-least squares-discriminant analysis (PLS-DA) was then evaluated. Finally, a tentative identification of the polyphenolic compounds characteristic for each honey variety under study was performed by LC-LRMS working in multiple-reaction monitoring (MRM) mode.

## 2. Results and Discussion

### 2.1. Off-Line SPE LC-LRMS Polyphenolic Fingerprints

**Figure 1:**
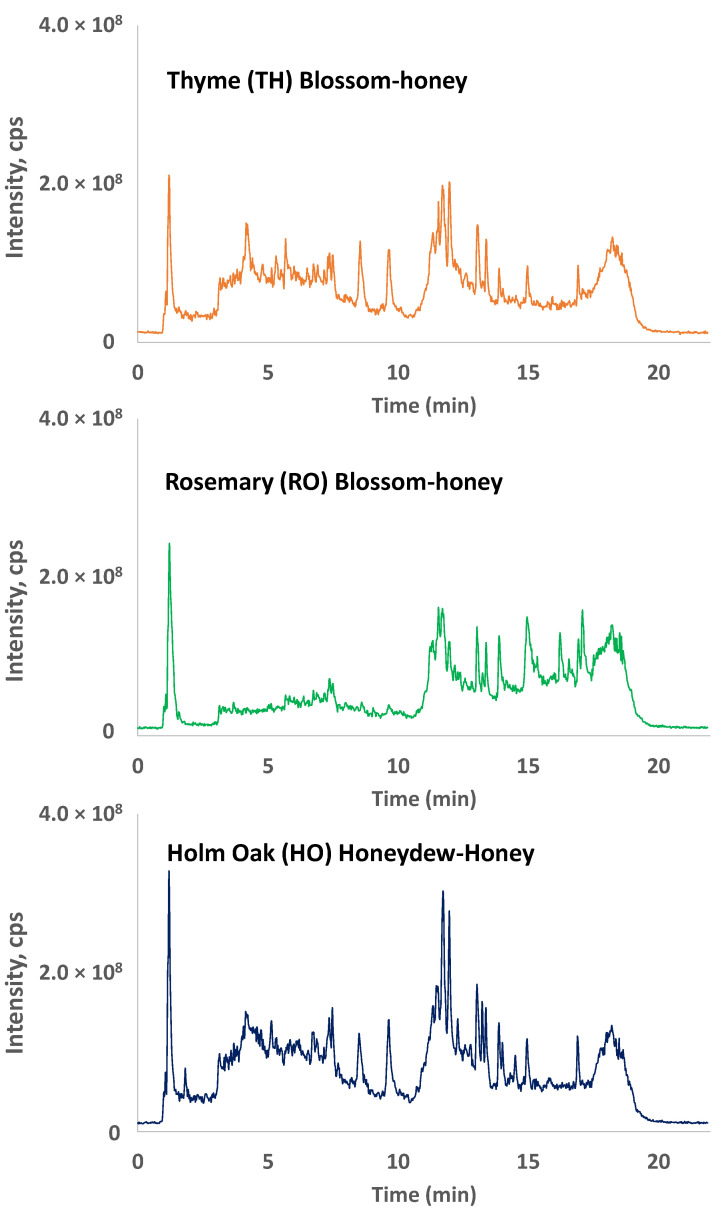
Off-line SPE HPLC-LRMS (total ion chromatogram) polyphenolic fingerprints for three selected honey samples.

In a previous contribution [34], an off-line solid-phase extraction (SPE) procedure optimized for the isolation of polyphenolic compounds using Oasis hydrophilic–lipophilic balanced (HLB) cartridges was developed. The obtained polyphenolic honey extracts were then analyzed by a HPLC-UV method employing a C18 reversed-phase separation and an optimized gradient elution program using 0.1% formic acid aqueous solution and acetonitrile as the mobile phase components (see Experimental Section 3.3). The UV fingerprints at 280 nm were discriminant enough to allow for the classification and differentiation between blossom and honeydew honeys as general classes, as well as within the different blossom-honey botanical varieties. However, the classification failed when dealing with the different honeydew-honey botanical varieties under study. The authentication of honey samples based on their geographical production region was deficient as well. Probably, one of the reasons for the low discrimination capability for some of the studied cases with HPLC-UV fingerprints is the low selectivity of UV detection. With the aim of obtaining better discriminant chemical descriptors, in the present work, the honey polyphenolic extracts were analyzed by reversed-phase (C18) liquid chromatography coupled to a QTrap hybrid triple-quadrupole/linear ion trap mass spectrometer and using an electrospray ionization (ESI) source working in negative ionization mode (ionization voltage of −2500 V). Data were acquired in enhanced full-scan mode (*m/z* 100–550) and, as a non-targeted polyphenolic fingerprinting strategy was intended, the total chromatographic signals were employed as sample chemical descriptors without the requirement of knowing the identity of the detected signals. As an example, the obtained fingerprints of three selected honey samples are depicted in Figure 1.

The obtained fingerprints present, in general, an important number of peak signals corresponding to substances isolated by SPE and detected by HPLC-LRMS. Most of these substances may correspond to polyphenolic and phenolic acid honey components (a tentative identification will be discussed later); however, in the present contribution, we intended to employ a fingerprinting strategy, in which the total ion chromatograms were employed as chemical descriptors to address sample classification. Hence, the identification of specific honey markers was not necessary. As can be seen, important differences corresponding to both the number of detected peaks and peak intensities are observed between the different honey types. As depicted in the figure, these differences are located mainly from 3.5 to 18 min. The number of substances detected in some of these time regions for some honey samples is scarce. This is, for example, the case of rosemary (RO) blossom honeys in the two first time segments, probably due to their low levels of phenolic compounds that typically elute at the beginning of the chromatogram in reversed-phase mode. In contrast, they are richer in peak signals detected at higher retention times, where typically flavonoid-based honey components elute. Other sample fingerprints, such as the ones depicted for thyme (TH) blossom honey and holm oak (HO) honeydew honey, are richer through the entire chromatogram, but important differences can be visually observed among them. Differences between the obtained off-line SPE HPLC-LRMS polyphenolic fingerprints and the fact that within a given honey botanical variety, the fingerprints are quite reproducible, encourage us to evaluate them as feasible sample chemical descriptors to address honey classification.

### 2.2. Exploratory Principal Component Analysis

**Figure 2:**
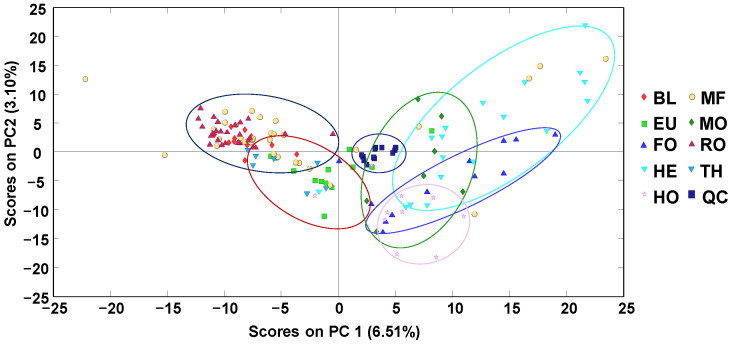
Score plot of PC1 vs. PC2 when using off-line SPE HPLC-LRMS polyphenolic fingerprints as honey chemical descriptors. BL: Orange/lemon blossom; EU: Eucalyptus; FO: Forest; HE: Heather; HO: Holm oak; MF: Multi-floral; MO: Mountain; RO: Rosemary; TH: Thyme; QC: Quality control.

As a first approach, off-line SPE HPLC-LRMS polyphenolic fingerprints were subjected to an exploratory (and non-supervised) chemometric method, such as principal component analysis (PCA), to evaluate the reproducibility of the proposed methodology analyzing the behavior of the QCs, as well as the distribution of the honey samples. As an example, Figure 2 depicts the obtained PCA score plot of PC1 vs. PC2.

As depicted in the figure, QCs (analyzed at the beginning of the sample sequence and after every 10 honey samples) appeared clustered close to the center of the PCA score plot, demonstrating the reproducibility and robustness of the proposed methodology and that the obtained chemometric results will not be affected by any sequence drift. Focusing on the honey samples, it can be observed that they tend to be grouped according to their botanical variety, although with important sample overlapping within some groups. In any case, blossom-honeys—located mainly in the left area of the score plot—and honeydew-honeys—located in the right area—could be easily distinguished. As an exception, blossom heather (HE) honeys are clustered in the same area as honeydew-honey samples. This behavior was also previously observed when employing non-targeted HPLC-UV fingerprinting methodologies [34]. Even though HE honeys are produced from the nectar of the plants, their properties and physicochemical characteristics are closer to those of honeydew honeys, all of them being characterized by their dark color and their higher total phenolic content (TPC) compared to other blossom honeys [1,2,18,36]. Certain discrimination in two groups with the contribution of both PC1 and PC2 values was also observed among the blossom honeys, with Rosemary (RO) and Orange/lemon blossom (BL) honeys showing mainly positive PC2 values, while Eucalyptus (EU) and Thyme (TH) honeys displayed negative PC2 values. This discrimination between RO/BL and EU/TH blossom honeys could be related to their different flavonoid composition, as previously reported in the literature [37]. Finally, multifloral (MF) honeys were spread through all the PCA score plot, although more concentrated in the area of blossom honeys.

### 2.3. Honey Classification Based on Botanical Variety by Partial Least Squares-Discriminant Analysis

**Figure 3:**
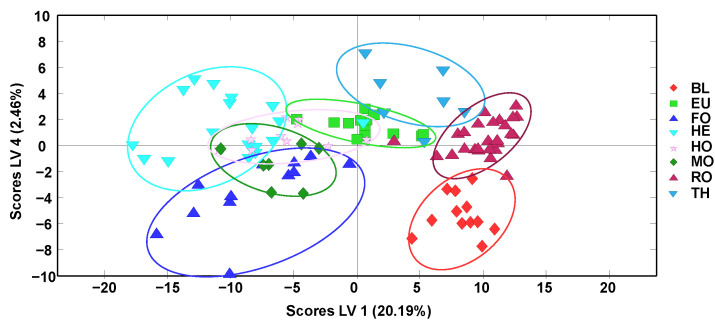
Partial least squares-discriminant analysis (PLS-DA) score plot of LV2 vs. LV4 when using off-line SPE HPLC-LRMS fingerprints as honey chemical descriptors (4 LVs were employed to build the model). BL: Orange/lemon blossom; EU: Eucalyptus; FO: Forest; HE: Heather; HO: Holm oak; MO: Mountain; RO: Rosemary; TH: Thyme.

The obtained off-line SPE HPLC-LRMS fingerprints were also subjected to supervised partial least squares-discriminant analysis (PLS-DA) for honey classification according to their botanical varieties. The scores plot of LV1 vs. LV4 is depicted in Figure 3. For simplification, QCs and multifloral (MF) honey samples were not considered. As expected, the discrimination between sample classes improved in comparison to the results obtained by PCA (Figure 2). Again, samples are grouped according to their botanical variety and, in general, a clear distinction between two groups of samples is observed: (i) BL, RO, EU, and TH blossom honeys (mainly exhibiting positive LV1 values) and (ii) HE blossom honey and MO, FO, and HO honeydew honeys (located to the left of the plot with negative LV1 values).

Better sample discrimination was accomplished among the five groups of blossom-honey samples (HE, BL, RO, EU, and TH), while honeydew honeys appeared overlapped between them and with both HE and EU blossom honeys. These results clearly improved those previously obtained with non-targeted HPLC-UV fingerprinting strategies [34] when considering together all the different honey botanical varieties under study, probably due to the higher selectivity of the HPLC-LRMS fingerprints.

**Figure 4:**
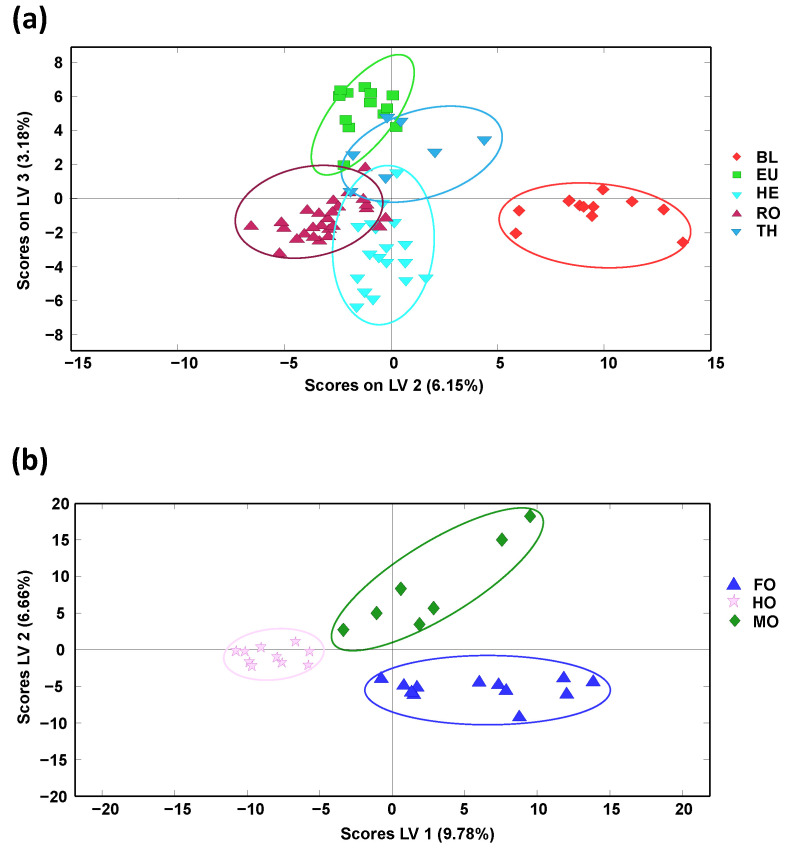
Partial least squares-discriminant analysis (PLS-DA) score plots when using off-line SPE HPLC-LRMS fingerprints as honey chemical descriptors for the classification of (**a**) honey-blossom botanical varieties (LV2 vs. LV3 score plot, 4 LVs were used to build the model) and (**b**) honeydew-honey botanical varieties (LV1 vs. LV2 score plot, 3 LVs were used to build the model). BL: Orange/lemon blossom; EU: Eucalyptus; FO: Forest; HE: Heather; HO: Holm oak; MO: Mountain; RO: Rosemary; TH: Thyme.

To study the classification capability of the proposed methodology, PLS-DA models by considering only blossom or honeydew honeys, independently, were also considered, and the obtained PLS-DA score plots are depicted in Figure 4.

When considering only blossom-honey botanical varieties (Figure 4a, depicting LV2 vs. LV3, being the two LVs showing the best discrimination), only the orange/lemon blossom (BL) variety was perfectly separated from the other four sample groups that, although grouped according to their botanical variety, showed some sample overlapping close to the center area of the plot. In any case, this discrimination was quite acceptable, as shown by the results of the multiclass prediction data by cross-validation, summarized in Table S1 (Supplementary Materials). In general, sensitivity and specificity values higher than 90% and 85%, respectively, were obtained, with classification errors below 12.4%, being BL honey class, the only group exhibiting 100% values for all evaluated parameters (sensitivity, specificity, and classification ratio), as expected. In contrast, perfect sample discrimination was observed when addressing only honeydew-honey botanical varieties (Figure 4b, depicting LV1 vs. LV2). Accordingly, the results observed in the multiclass prediction data by cross-validation (data summarized in Table S2 in Supplementary Materials) showed 100% values for both sensitivity and specificity and a ratio of classification within each sample group also of 100%, results that exceed the performance previously described using off-line SPE HPLC-UV polyphenolic fingerprints as honey chemical descriptors [34].

### 2.4. Honey Classification Based on Geographical Climatic Production Regions by Partial Least Squares-Discriminant Analysis

**Figure 5:**
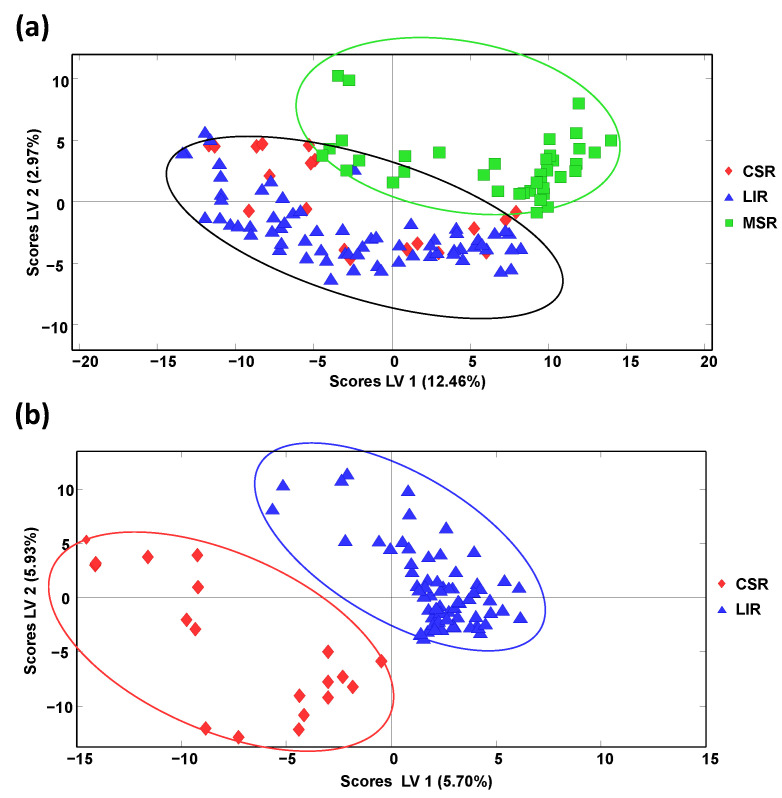
Partial least squares-discriminant analysis (PLS-DA) score plots when using off-line SPE HPLC-LRMS fingerprints as honey chemical descriptors for the classification (**a**) based on their climatic geographical production regions (LV1 vs. LV2 score plot, 2 LVs were used to build the model) and (**b**) CSR and LIR honey samples (LV1 vs. LV2 score plot, 2 LVs were used to build the model). CSR: Cantabrian Sea region; LIR: Landlock Inland region; and MSR: Mediterranean Sea region.

Honey classification focused on production region was also evaluated by PLS-DA, employing the obtained off-line SPE HPLC-LRMS polyphenolic chromatographic fingerprints as sample chemical descriptors. It should be mentioned that sample discrimination according to the different Spanish geographical production regions described in Table S3 (Supplementary Materials) was not accomplished, as previously described when using HPLC-UV fingerprints [34], probably due to the proximity between many of these geographical areas. For this reason, bigger geographical production regions based on Spanish climatic conditions were considered, mainly the continental climatic area (landlocked inland region, LIR) and sea climatic areas, considering, in this case, the Cantabrian Sea region (CSR), at the North coast of Spain, and the Mediterranean Sea region (MSR), at the East/South coast of Spain. The samples employed within each established geographical climatic region are also indicated in Table S3. Samples labelled as Spain and Spain and others were not considered. When evaluating the proposed off-line SPE HPLC-LRMS polyphenolic fingerprints as sample chemical descriptors to accomplish this sample discrimination by PLS-DA, only honey samples belonging to the Mediterranean Sea region were clearly differentiated from the other two groups that appeared overlapped, as can be observed in Figure 5a showing the PLS-DA score plot of LV1 vs. LV2.

Nevertheless, a paired PLS-DA model employing only the samples belonging to the LIR and CSR groups (Figure 5b) showed perfect discrimination of these two honey sample groups, demonstrating that the proposed fingerprints were feasible to classify the honey samples under study according to their climatic geographical production region, at least when employing a sequential PLS-DA classification model.

### 2.5. Honey Polyphenolic Identification by LC-MS/MS in Multiple-Reaction Monitoring (MRM) Mode

**Figure 6:**
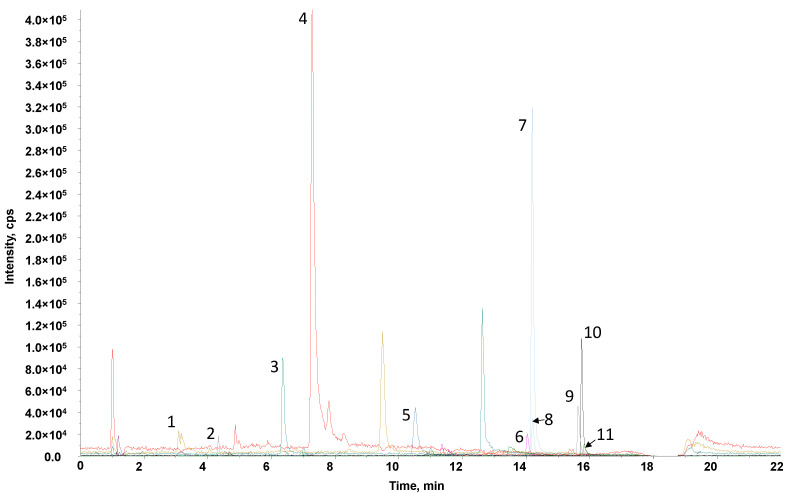
Extracted ion chromatogram of a multifloral honey off-line SPE sample extract analyzed by LC-MS/MS in MRM mode (acquisition conditions are described in Table S4 in Supplementary Materials). Peak identification: 1, gallic acid; 2, 3-hydroxytyrosol; 3, 4-hydroxybenzoic acid; 4, caffeic acid; 5, *p*-coumaric acid; 6, apigenin; 7, pinobanksin; 8, narigenin; 9, chrysin; 10, pinocembrin; and 11, galangin.

The diversity of polyphenolic compounds that can be found in honey is a remarkable factor, since they define various properties, such as the antioxidant capacity. In the present work, a tentative identification of polyphenolic compounds in the different honey botanical varieties was carried out. The extracts obtained by off-line SPE were analyzed with a liquid chromatography-tandem mass spectrometry (LC-MS/MS) method, monitoring a total of 53 polyphenolic compounds (Table S4, Supplementary Materials) in MRM acquisition mode. As an example, Figure 6 shows the extracted ion chromatogram of the transitions of the most notable polyphenolic compounds detected in a multifloral honey sample. For simplification, only the most abundant detected phenolic compounds are depicted in the figure.

Table 1 summarizes the identified polyphenolic compounds according to the honey botanical variety. It should be pointed out that, at this level, we were not interested in the quantification of these compounds but only in their presence in the analyzed samples to select polyphenolic compounds as sample markers for future studies. In addition, when a phenolic compound is labelled in the table as not detected (marked in red(-)), it means that it was not detected in any of the analyzed samples within the same sample group. In contrast, when a phenolic compound is labelled as detected (marked in green(+)), it means that, on average, it was detected in most of the analyzed samples within the same sample group, although it was not present in some specific samples.

Among the 53 monitored polyphenolic compounds, 18 of them (caftaric acid, *trans*-coumaric acid, astilbin, catechin, epicatechin, hesperetin, myricetin, procyanidin A2, B2 and C1, polydatin, resveratrol, 3-methylcatechol, 4-methylcatechol, catechol, epigallocatechin, ethyl gallate, and oleuropein) were not detected in any of the analyzed honey samples, while only 8 of them (3,4-dihydroxybenzoic acid, 4-hydroxybenzoic acid, caffeic acid, gallic acid, *p*-coumaric acid, pinobanksin, rutin, and 3-hydroxytyrosol) were detected in all the botanical varieties. Some compounds were detected only in a specific honey botanical variety. This is the case, for example, of naringin, which was only detected in the orange/lemon blossom (BL) botanical variety or vanillin in the blossom-multifloral (MF) varieties. In the first case, naringin, a characteristic flavonoid found in the peel and pulp of citrus fruits, becomes a specific flavonoid marker of BL samples. The second case is not so clear, as the presence of vanillin only in the MF samples analyzed in this study could be due to other different botanical varieties (not considered here). Other compounds were found in MF and another botanical variety, such as the case of syringic acid (found in MF and thyme samples) or galangin (found in MF and rosemary samples), thus, becoming marker candidates for TH and RO blossom-honey samples, respectively. However, this needs to be confirmed in future studies as galangin has been reported as a typical flavanol found in propolis [38], which would explain its presence, in general, in any honey sample. More studies using high-resolution mass spectrometry for the unequivocal identification of the proposed markers and employing a high number of honey samples will be required in order to ensure the assignment of a specific phenolic compound as a marker of a given honey variety.

As can be seen, the number of phenolic acids found in the different honey botanical varieties under study is quite similar, ranging from six (in BL samples) to nine (in MO and RO samples). In contrast, flavonoids are more abundant in some botanical varieties, such as MF, followed by RO and EU samples, and MO and TH samples.

In any case, further studies will be necessary to confirm the possibility of proposing specific polyphenolic compounds as markers for the authentication of honey samples.

## 3. Materials and Methods

### 3.1. Reagents and Chemicals

Methanol (Chromosolv^TM^ for HPLC, ≥99.9%) and acetonitrile (UHPLC supergradient ACS quality) were obtained from PanReac AppliChem (Barcelona, Spain), formic acid (≥98%) from Sigma-Aldrich (St Louis, MO, USA), and hydrochloric acid (37%) from Fisher Chemical (Geel, Belgium). Water was purified by employing an Elix 3 system coupled to a Milli-Q instrument from Millipore Corporation (Bedford, MA, USA). The water was filtered with a 0.22 µm nylon membrane filter integrated into the Milli-Q instrument.

All the polyphenolic and phenolic acid compounds used in this work were of analytical grade and were obtained from Sigma-Aldrich, with the exception of hesperidin obtained from Glentham Life Sciences (Lorsham, United Kingdom), astilbin and caftaric acid from Biopurity Phytochemicals Ltd. (Chengdu, Sichuan, China), trans-coumaric acid and procyanidin C1 from Phytolab (Vestenbergsgreuth, Germany), diosmin, hesperetin, and catechol from AlfaAesar Chemicals (ThermoFisher, Kandel, Germany), naringin, naringenin, and epigallocatechin from Biosynth-Carbosynth (Berkshire, United Kingdom), procyanidins B2 and A2 from Extrasynthese (Genay, France), pinocembrin from Fisher Scientific (Madrid, Spain), tricetin and galangin from Cymit Quimica S.L. (Barcelona, Spain), and chrysin and pinobanksin from Merck (Darmstadt, Germany).

### 3.2. Samples and Sample Treatment

The analyzed Spanish honey samples are listed in Table S3 (Supplementary Materials). All the honey samples were obtained from supermarkets and local markets in Spain, and included 34 multifloral (MF) honeys and 102 monofloral honeys of which 76 were blossom honeys of different botanical origins: orange/lemon blossom (BL), rosemary (RO), thyme (TH), Eucalyptus (EU), and heather (HE); 26 were honeydew honeys of different botanical origins: mountain (MO), forest (FO), and holm oak (HO). Two heather honeys were donated by Miel de Braña (León, Spain). Among the different honey botanical varieties, samples from different Spanish geographical origins were analyzed, among them honeys obtained from the regions of Asturias (AS), Aragon (AR), Basque Country (BC), Cantabria (CN), Castile La Mancha (CM), Castile and Leon (CL), Catalonia (CT), Extremadura (E), Balearic Islands (BI), and Navarre (N), as well as samples labeled as Spain (S), where the Spanish geographical region was not indicated, and Spain and others (SO), which include mixtures of honeys produced in Spain with that of other countries such as Uruguay, Cuba, Mexico, Romania, or Ukraine.

The isolation and preconcentration of polyphenolic compounds from honey samples was performed following a previously developed off-line SPE procedure [34]. Briefly, ~1 g of honey was dissolved in 10 mL of 0.01 MHCl (pH 2.0) in a 15 mL PTFE tube (Serviquimia, Barcelona, Spain) by vigorously shaking in a Vortex (VibraMix, Barcelona, Spain). Oasis hydrophilic–lipophilic balanced (HLB, 3 cc, 60 mg) cartridges from Waters (Milford, MA, USA), previously conditioned with 2 mL of methanol and 3 mL of acidified water (0.01 M HCl), were employed for the SPE procedure. The 10 mL of the aqueous honey extracts was loaded into the HLB SPE cartridges (flow rate of 1–2 mL min^−1^) by employing a Visiprep system (Supelco, Bellefonte, PA, USA). Then, the cartridges were cleaned with 2 mL of acidified water (0.01 M HCl) and dried with air. Elution was performed with 6 mL of methanol, which was collected into 15 mL PTFE tubes. The eluates were evaporated to dryness in a water bath at 40 °C and under a nitrogen stream using a TurboVap LV evaporator (Caliper model, Marshall Scientific, Hampton, NH, USA). The residues were dissolved in 1 mL of water:acetonitrile (95:5 *v/v*), filtered using 0.45 µm syringe membrane filters (FILTER-LAB, Barcelona, Spain), and stored in 2 mL amber chromatographic injection vials at 4 °C until LC-LRMS analysis.

A quality control (QC) sample was prepared by mixing 50 µL of each one of the re-constituted polyphenolic honey extracts. This QC was employed to assess the reproducibility of the proposed HPLC-LRMS fingerprinting methodology and to ensure the robustness of the chemometric results.

### 3.3. HPLC-LRMS Method

Polyphenolic extracts were analyzed in an Agilent 1100 Series HPLC instrument (Waldbronn, Germany) using a Kinetex^®^ C18 porous-shell (100 × 4.6 mm I.D., 2.6 µm partially porous particle size) column (Phenomenex, Torrance, CA, USA) under gradient elution following a previously proposed chromatographic separation method [39]. Then, 0.1% formic acid aqueous solution and acetonitrile were used as mobile phase components following the gradient elution program indicated in Table 2. The injection volume was 5 µL.

The HPLC instrument was coupled to an AB Sciex 4000 QTrap hybrid triple-quadrupole/linear ion trap mass spectrometer (Framingham, MA, USA) using an electrospray ionization (ESI) source working in negative ionization mode. Ion spray voltage and source temperature were set at −2500 V and 400 °C, respectively. Nitrogen was used for the curtain gas, ion source gas 1, and ion source gas 2 at 10, 50, and 50 arbitrary units (a.u.), respectively. A declustering potential (DP) of −80 V was selected. HPLC-LRMS fingerprints were registered in enhanced full-scan mode (*m/z* 100–550).

For the tentative identification of polyphenolic compounds in honey samples, LC-LRMS in multiple-reaction monitoring (MRM) acquisition mode was used. The list of the 53 polyphenolic compounds monitored as well as the acquisition conditions (precursor ion, product ion, collision energy) are summarized in Table S4 (Supplementary Materials). Analyst software version 1.6.2 (AB Sciex, Framingham, MA, USA) was used to control the LC-LRMS system and data processing.

### 3.4. Data Processing

#### 3.4.1. Data Matrix Building

MSConvert free software (ProteoWizard, Palo Alto, CA, USA) was employed to convert the chromatographic raw data into an mzML output format [39,40]. For that purpose, a binary encoding precision of 32 bits and a Threshold Peak Filter (absolute intensity as threshold type of 10,000 counts) were employed.

Next, the obtained mzML files were transformed into a data matrix containing the off-line SPE LC-LRMS polyphenolic fingerprints (in which the ion signal intensities are arranged as a function of the samples and the variables in rows and columns, respectively) by employing the free mzMine 3 software (version 17.0.2) [41]. With this aim, mass lists for each of the scans registered in a given sample (by considering 1.5 × 10^4^ as the noise level) were created by means of the exact mass-detection feature. Then, false signals were removed by employing the FTMS shoulder peak filter after setting a Gaussian peak model function and 70,000 as mass resolution. Next, the exact mass signals found in contiguous scans of a given sample were joined by the automated data analysis pipeline (ADAP) chromatogram builder. For that purpose, 0.5–22 min, 5 ppm, and 1.5 × 10^4^ were set as peak time range, *m/z* tolerance, and intensity threshold, respectively. Isotopes were removed considering the most intense one as the most representative isotope and setting an *m/z* of 1 as the mass tolerance. Then, to match the detected masses across all the analyzed honey samples, the Join Aligner feature was employed by setting 5 ppm, 90%, 2 min, and 1% as the values for the mass tolerance, the weight for *m/z*, the retention time tolerance, and the weight for the retention time, respectively. Finally, the aligned feature list obtained was exported as CSV format. The data matrix obtained had a dimension (samples + QCs × variables) of (151 × 1436), using as variables the signal detected for an ion with a specific *m/z* and eluting at a specific retention time.

#### 3.4.2. Chemometric Data Analysis

Principal component analysis (PCA) and partial least squares-discriminant analysis (PLS-DA) methods were from SOLO 8.6 chemometric software (Eigenvector Research, Manson, WA, USA). Theoretical aspects and more information about these chemometric methods can be found in [42].

Non-supervised PCA was employed with the aim of exploring the analyzed honey sample distribution and the behavior of the QC solution. Then, PLS-DA was applied for classification purposes according to both honey botanical varieties and geographical production regions. The employed X-data matrix for PCA consisted of the off-line SPE LC-LRMS polyphenolic fingerprints for each honey and QC sample (i.e., the signal intensity values of all the ions detected as a function of *m/z* and retention time). The same X-data matrix but without QCs was used for PLS-DA, while the Y-data matrix defined the sample classes, i.e., the honey botanical variety or geographical production region, depending on the evaluated case. The number of latent variables (LVs) used in PLS-DA was estimated by the first relevant minimum of the cross-validation (CV) error from a Venetian blind approach.

## 4. Conclusions

In this work, off-line SPE LC-LRMS polyphenolic fingerprints were evaluated as sample chemical descriptors for the classification and authentication of Spanish honey samples by multivariate chemometric methods. In general, the proposed fingerprints showed a very good classification and discrimination capacity by PLS-DA to differentiate the different Spanish blossom- and honeydew-honey botanical varieties under study. Highly satisfactory cross-validation multiclass prediction values were achieved for the set of blossom honey samples: sensitivity and specificity higher than 90% and 85%, respectively, and classification errors below 12.4%. Perfect discrimination was accomplished for the set of honeydew-honey samples, with a 100% sensitivity, specificity, and classification ratio, clearly improving results reported previously using HPLC-UV fingerprints, where no discrimination at all was observed for this group of samples.

The proposed fingerprints were also appropriate to characterize and classify the analyzed honey samples according to the climatic production region, being able to distinguish honeys from the Mediterranean Sea region versus the others and with a second paired PLS-DA model that discriminated between Cantabrian Sea and landlocked inland regions.

The results obtained in this work allow one to propose the off-line SPE LC-LRMS polyphenolic fingerprinting strategy as a feasible methodology to address the characterization, classification, and authentication of Spanish honey samples based on their different botanical and climatic region attributes.

Finally, the proposed methodology was also employed for the tentative identification of polyphenolic compounds according to the different botanical varieties under study. For that purpose, tandem mass spectrometry in multiple-reaction monitoring mode was employed. Phenolic acids were present in all the analyzed samples. In contrast, flavonoids were more abundant in botanical varieties, such as rosemary, eucalyptus, mountain, and thyme honeys. Some polyphenolic compounds were characteristic of some varieties, such as naringin, syringic acid, and galangin, specific for orange/lemon blossom, thyme, and rosemary varieties, respectively. These compounds could be proposed as tentative honey markers to solve authentication issues, although more studies will be required to confirm this hypothesis.

## Figures and Tables

**Table 1:** Summary of tentative identified and detected polyphenolic compounds in the honey samples according to their botanical variety. BL: Orange/lemon blossom; EU: Eucalyptus; FO: Forest; HE: Heather; HO: Holm oak; MF: Multi-floral; MO: Mountain; RO: Rosemary; TH: Thyme. Detected compounds are marked in green (+), and non-detected compounds in red (-).

Family	Compound	MF	MO	HE	RO	EU	FO	TH	BL	HO
Phenolic acids	2,5-dihydroxybenzoic acid	-	-	+	-	+	+	-	-	-
	3,4-dihydroxybenzoic acid	+	+	+	+	+	+	+	+	+
	4-hydroxybenzoic acid	+	+	+	+	+	+	+	+	+
	Caffeic acid	+	+	+	+	+	+	+	+	+
	Caftaric acid	-	-	-	-	-	-	-	-	-
	Chlorogenic acid	-	+	+	+	-	-	-	-	+
	Ferulic acid	+	+	+	+	+	+	-	-	-
	Gallic acid	+	+	+	+	+	+	+	+	+
	*p*-Coumaric acid	+	+	+	+	+	+	+	+	+
	Quinic acid	-	+	-	+	-	-	-	-	-
	Syringic acid	+	-	-	-	-	-	+	-	-
	*trans*-Coutaric acid	-	-	-	-	-	-	-	-	-
	Vanillic acid	+	+	-	+	+	+	+	+	+
Flavonoids	Apigenin	+	-	-	+	+	-	-	-	-
	Astilbin	-	-	-	-	-	-	-	-	-
	Catechin	-	-	-	-	-	-	-	-	-
	Chrysin	+	+	-	+	+	-	+	-	-
	Diosmin	+	+	-	+	+	-	+	+	+
	Epicatechin	-	-	-	-	-	-	-	-	-
	Galangin	+	-	-	+	-	-	-	-	-
	Hesperetin	-	-	-	-	-	-	-	-	-
	Hesperidin	+	-	-	+	+	-	+	+	+
	Kaempferol	+	-	-	-	-	-	-	-	-
	Luteonin	+	-	-	+	+	-	-	-	-
	Myricetin	-	-	-	-	-	-	-	-	-
	Naringenin	+	+	+	+	+	+	+	-	-
	Naringin	-	-	-	-	-	-	-	+	-
	Pinobanksin	+	+	+	+	+	+	+	+	+
	Pinocembrin	+	+	+	+	+	+	+	-	-
	Procyanidin A2	-	-	-	-	-	-	-	-	-
	Procyanidin B2	-	-	-	-	-	-	-	-	-
	Procyanidin C1	-	-	-	-	-	-	-	-	-
	Quercetin	+	-	-	+	+	-	-	-	-
	Rutin	+	+	+	+	+	+	+	+	+
	Tricetin	-	+	+	-	+	-	-	-	-
Stilbenes	Polidatin	-	-	-	-	-	-	-	-	-
	Resveratrol	-	-	-	-	-	-	-	-	-
Other polyphenols	3-hydroxytyrosol	+	+	+	+	+	+	+	+	+
	3-methylcatechol	-	-	-	-	-	-	-	-	-
	4-ethylcatechol	+	+	+	+	+	+	-	+	+
	4-methylcatechol	-	-	-	-	-	-	-	-	-
	Catechol	-	-	-	-	-	-	-	-	-
	Epigalocatechin	-	-	-	-	-	-	-	-	-
	Ethyl gallate	-	-	-	-	-	-	-	-	-
	Oleuropein	-	-	-	-	-	-	-	-	-
	Vanillin	+	-	-	-	-	-	-	-	-

**Table 2:** Gradient elution conditions.

Time (min)	Elution	% Acetonitrile	Flow Rate (µL min^−1^)
0–1	Linear gradient	5–10%	800
1–4	Linear gradient	10–16%	800
4–8	Isocratic	16%	800
8–8.5	Linear gradient	16–25%	800
8.5–13.5	Linear gradient	25–60%	800
13.5–16	Linear gradient	60–100%	800
16–16.5	Isocratic	100%	800
16.5–16.6	Linear gradient	100–5%	800
16.6–22	Isocratic	5%	800

## Data Availability

Data are available upon request to the authors.

## Supplementary Materials

The following supporting information can be downloaded at: https://www.mdpi.com/article/10.3390/molecules27227812/s1, Table S1: Multiclass predictions by cross-validation for the set of blossom-honey samples using 3 LVs. BL: Orange/lemon blossom; RO: Rosemary; EU: Eucalyptus; TH: Thyme, and HE: Heather; Table S2: Multiclass predictions by cross-validation for the set of honeydew-honey samples using 3 LVs. HO: Holm oak; MO: Mountain; and FO: Forest; Table S3: Number of analyzed honey samples considering botanical varieties and geographical origins; Table S4: Monitored polyphenolic compounds and MRM acquisition conditions.

## Sample Availability

Samples are available upon request to the authors.
