# Supplementary material for: Off-Line SPE LC-LRMS Polyphenolic Fingerprinting and Chemometrics to Classify and Authenticate Spanish Honey
Source: Molecules. 2022 Nov 13;27(22):7812. doi: 10.3390/molecules27227812 (PMC9695661; doi:10.3390/molecules27227812)
Supplement: Supplementary file 1 [file molecules-27-07812-s001.zip › molecules-2021347-supplementary.pdf]

Supplementary Material

Off-line SPE LC-LRMS Polyphenolic Fingerprinting and Chemometrics to Classify and Authenticate Spanish Honey

Víctor García-Seval [1], Javier Saurina [1,2], Sònia Sentellas [1,2,3] and Oscar Núñez [1,2,3,*]

[1] Department of Chemical Engineering and Analytical Chemistry, University of Barcelona, Martí i Franquès 1-11, E08028 Barcelona, Spain

[2] Research Institute in Food Nutrition and Food Safety, University of Barcelona, Recinte Torribera, Av. Prat de la Riba 171, Edifici de Recerca (Gaudí), Santa Coloma de Gramenet, E08921 Barcelona, Spain

[3] Serra Húnter Fellow, Generalitat de Catalunya, Rambla de Catalunya 19-21, E-08007 Barcelona, Spain

* Correspondence: oscar.nunez@ub.edu

Table S1. Multiclass predictions by cross-validation for the set of blossom-honey samples using 3 LVs. BL: Orange/lemon blossom; RO: Rosemary; EU: Eucalyptus; TH: Thyme, and HE: Heather.

Sample class variety	Sensitivity (%)	Specificity (%)	Classification Error (%)
BL	100	100	0
EU	95.4	90.1	10.5
HE	92.2	86.4	11.6
RO	91.3	85.4	10.2
TH	90.4	93.1	12.4

Table S2. Multiclass predictions by cross-validation for the set of honeydew-honey samples using 3 LVs. HO: Holm oak; MO: Mountain; and FO: Forest.

Sample class variety	Sensitivity (%)	Specificity (%)	Classification Error (%)
HO	100	100	0
MO	100	100	0
FO	100	100	0

Table S3. Number of analyzed honey samples considering botanical varieties and geographical origins

Honey	Botanical variety	Number of samples	Geographical origin	Number of samples	Climatic geographical origin
Blossom-honeys	Orange/Lemon Blossom (BL)	12	Aragon	1	LIR
			Balearic Islands	2	MSR
			Cantabria	1	CSR
			Castile and Leon	2	LIR
			Catalonia	3	MSR
			Extremadura	1	LIR
			Spain[a]	2	-
	Eucalyptus (EU)	13	Andalusia	1	MSR
			Asturias	2	CSR
			Cantabria	1	CSR
			Castile and Leon	2	LIR
			Catalonia	1	MSR
			Extremadura	4	LIR
			Navarre	1	CSR
			Spain and others[a]	1	-
	Rosemary (RO)	26	Andalusia	1	MSR
			Aragon	4	LIR
			Asturias	1	CSR
			Balearic Islands	4	MSR
			Castile and Leon	1	LIR
			Cantabria	2	CSR
			Catalonia	6	MSR
			Extremadura	4	LIR
			Navarre	1	CSR
			Spain[a]	2	-
	Thyme (TH)	7	Castile and Leon	1	LIR
			Castile La Mancha	2	LIR
			Catalonia	1	MSR
			Extremadura	2	LIR
			Spain[a]	1	-
	Heather (HE)	18	Asturias	2	CSR
			Basque Country	1	CSR
			Cantabria	3	CSR
			Castile and Leon	5	LIR
			Catalonia	2	MSR
			Extremadura	5	LIR
Honeydew-honeys	Mountain (MO)	6	Asturias	2	CSR
			Castile and Leon	1	LIR
			Castile La Mancha	1	LIR
			Catalonia	2	MSR
	Forest (FO)	10	Balearic Islands	2	MSR
			Cantabria	2	CSR
			Castile and Leon	1	LIR
			Catalonia	1	MSR
			Spain[a]	4	-
	Holm Oak (HO)	10	Aragon	2	LIR
			Castile and Leon	2	LIR
			Extremadura	6	LIR
Other honeys	Multifloral (MF)	34	Asturias	1	CSR
			Balearic Islands	4	MSR
			Cantabria	1	CSR
			Castile and Leon	7	LIR
			Castile La Mancha	2	LIR
			Catalonia	8	MSR
			Extremadura	4	LIR
			Navarre	4	CSR
			Spain[a]	1	-
			Spain and others[a]	2	-

[a] *Spain: Honeys produced in Spain but geographical region not specified; Spain and others: Honeys that include mixtures of honey produced in Spain and other countries such as Uruguay, Cuba, Mexico, Romania or Ukraine*

Table S4. Monitored polyphenolic compounds and MRM acquisition conditions.

Polyphenolic compound	Retention time (min)	Precursor ion (*m/z*)	Product ion (*m/z*)	Declustering potential (DP, V)	Collision energy (CE, V)	Cell exit potential (CXP, V)
Gallic acid	3.1	169	125	-40	-22	-19
Caffeic acid	7.3	179	135	-45	-20	-1
Quercetin	12.6	301	151	-80	-32	-1
Hesperidin	11.8	609	301	-115	-36	-19
Resveratrol	13.0	227	143	-70	-34	-1
Ferulic acid	11.3	193	134	-30	-22	-1
Vanillic acid	7.0	167	152	-65	-20	-1
Ethyl gallate	9.5	197	124	-60	-34	-3
Catechin	6.3	289	109	-110	-42	-19
Epicatechin	7.5	289	125	-85	-33	-7
p-Coumaric acid	6.7	163	119	-60	-18	-1
Rutin	11.3	609	300	-95	-48	-23
Myricetin	12.9	317	151	-95	-40	-9
Syringic acid	7.2	197	121	-40	-26	-9
Astilbin	11.6	449	285	-100	-32	-1
trans-Coutaric acid	6.7	295	163	-30	-20	-11
Caftaric acid	5.4	311	179	-50	-22	-11
Diosmin	11.8	607	299	-105	-34	-23
Hesperetin	11.8	301	286	-105	-36	-5
Naringin	11.8	579	271	-140	-36	-5
Naringenin	14.2	271	151	-90	-32	-25
Catechol	5.8	109	91	-25	-26	-5
4-Hydroxybenzoic acid	6.3	137	93	-55	-18	-7
Ellagic acid	14.2	301	284	-95	-98	-1
Vanillin	9.0	151	136	-50	-14	-31
Epigallocatechin	5.6	305	125	-80	-26	-25
Chlorogenic acid	6.1	353	191	-60	-20	-17
3-Methylcatechol	11.0	123	108	-50	-22	-13
4-Ethylcatechol	12.7	137	122	-80	-22	-5
2,5-Dihydroxibenzoic acid	6.6	153	108	-50	-18	-15
4-Methylcatechol	11.0	123	108	-75	-22	-7
3,4-Dihydroxybenzoic acid	4.7	153	109	-70	-20	-3
Procyanidin B2	7.0	577	407	-5	-26	-11
Procyanidin A2	11.7	575	285	-135	-38	-1
Procyanidin C1	10.9	865	125	-180	-86	-7
3-Hydroxytyrosol	7.2	153	123	-50	-18	-5
Kaempferol	11.6	285	151	-85	-26	-7
Apigenin	11.7	269	117	-95	-48	-7
Luteolin	13.3	285	133	-95	-50	-7
Oleuropein	11.6	539	59	-60	-78	-11
4-Vinylguiacol	12.6	149	121	-125	-16	-9
trans-Cinnamic acid	12.8	147	103	-50	-16	-17
Guaiacol	9.4	123	95	-160	-20	-15
Pyrogallol	1.4	125	79	-10	-6	-1
Quinic acid	6.1	191	85	-80	-30	-5
Polydatin	13.5	389	227	-85	-20	-19
3,4-di-O-Caffeoylquinic acid	10.7	515	353	-75	-28	-25
4,5-di-O-Caffeoylquinic acid	11.8	515	173	-105	-36	-1
Pinocembrin	15.7	255	151	-65	-38	-1
Tricetin	12.6	301	149	-75	-52	-25
Galangin	15.8	269	41	-80	-100	-3

Chrysin	15.6	253	63	-90	-46	-3
Pinobanksin	14.2	271	253	-75	-32	-11
